# Indoor versus outdoor transmission of SARS-COV-2: environmental factors in virus spread and underestimated sources of risk

**DOI:** 10.1007/s41207-021-00243-w

**Published:** 2021-02-10

**Authors:** Vincenzo Senatore, Tiziano Zarra, Antonio Buonerba, Kwang-Ho Choo, Shadi W. Hasan, Gregory Korshin, Chi-Wang Li, Mohamed Ksibi, Vincenzo Belgiorno, Vincenzo Naddeo

**Affiliations:** 1 Sanitary Environmental Engineering Division (SEED), Department of Civil Engineering, University of Salerno, Via Giovanni Paolo II, Fisciano, Salerno Italy; 2 Inter-University Centre for Prediction and Prevention of Relevant Hazards (C.U.G.RI.), Via Giovanni Paolo II, Fisciano, Salerno Italy; 3 Department of Environmental Engineering, Kyungpook National University (KNU), 80 Daehak-ro, Buk-gu, Daegu, 41566 Republic of Korea; 4 Center for Membranes and Advanced Water Technology (CMAT), Department of Chemical Engineering, Khalifa University of Science and Technology, P.O. Box 127788, Abu Dhabi, United Arab Emirates; 5 Department of Civil and Environmental Engineering, University of Washington, P.O. Box 352700, Seattle, WA 98105-2700 USA; 6 Department of Water Resources and Environmental Engineering, Tamkang University, 151 Yingzhuan Road Tamsui District, New Taipei City, 25137 Taiwan; 7 Laboratoire de Génie de L’Environnement et Ecotechnologie, GEET-ENIS, Université de Sfax, Route de Soukra km 4, P.O. Box 1173, 3038 Sfax, Tunisia

**Keywords:** COVID-19, SARS-CoV-2, Transmission pathways, Aerosolized particle, Ventilation system, Wastewater, Air pollution

## Abstract

The first case of Coronavirus Disease 2019 (COVID-19), which is caused by Severe Acute Respiratory Syndrome Coronavirus 2 (SARS-CoV-2), in Europe was officially confirmed in February 2020. On 11 March 2020, after thousands of deaths from this disease had been reported worldwide, the WHO changed their classification of COVID-19 from a public health emergency of international concern to a pandemic. The SARS-CoV-2 virus has been shown to be much more resistant to environmental degradation than other coated viruses. Several studies have shown that environmental conditions can influence its viability and infectivity. This review summarizes current knowledge on the transmission pathways of the novel coronavirus, and directs attention towards potentially underestimated factors that affect its propagation, notably indoor spread and outdoor risk sources. The contributions of significant indoor factors such as ventilation systems to the spread of this virus need to be carefully ascertained. Outdoor risk sources such as aerosolized particles emitted during wastewater treatment and particulate matter (PM), both of which may act as virus carriers, should be examined as well. This study shows the influence of certain underestimated factors on the environmental behavior and survival of the SARS-CoV-2 virus. These aspects of coronavirus propagation need to be accounted for when devising actions to limit not only the current pandemic but also future outbreaks.

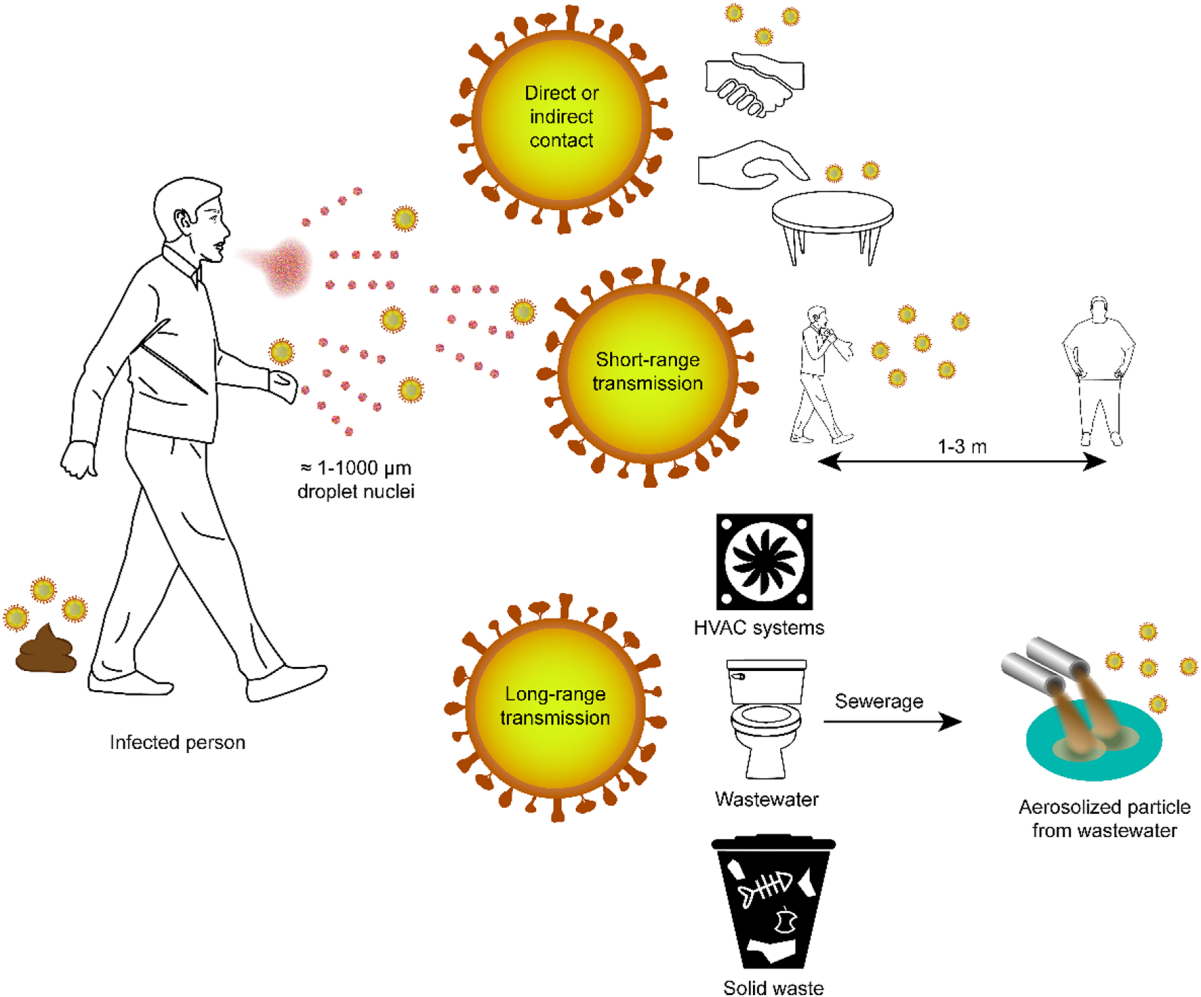

## Introduction

Severe Acute Respiratory Syndrome (SARS) and Middle East Respiratory Syndrome (MERS)—which are caused by the two coronaviruses SARS-CoV and MERS-CoV, respectively—are responsible for large-scale respiratory disease outbreaks that have led to the deaths of thousands of people (Raoult et al. 2020). SARS was identified in China 18 years ago, while MERS was first discovered 8 years ago in Saudi Arabia (Wang et al. 2020a, b). These viruses are highly pathogenic to humans and are believed to have originated from bats. In December 2019, an outbreak of pneumonia in Wuhan (China) attracted tremendous attention due to the rapid spread of this disease worldwide. The then-unknown cause of the pneumonia (later called COVID-19) has since been identified as the novel coronavirus SARS-CoV-2. This virus shares only 79.6% of its gene sequence with SARS-CoV (Zhou et al. 2020). In 2019, Fan et al. (2019) seemed to predict the pandemic declared by the World Health Organization (WHO) on 11 March 2020, stating that: “It is highly likely that future SARS- or MERS- like coronavirus outbreaks will originate from bats, and there is an increased probability that this will occur in China. Therefore, the investigation of bat coronaviruses becomes an urgent issue for the detection of early warning signs, which in turn minimizes the impact of such future outbreaks in China” (Fan et al. 2019).

The novel coronavirus has infected many more people than MERS and SARS have (Guarner 2020; Velavan and Meyer 2020; Wang et al. 2020a, b). Various factors have contributed to the rapid spread of COVID-19. At the initial phase of the pandemic, these included the high density of transport connections between Wuhan (one of the principal transportation hubs in China) and the rest of the world, increasing the chance of person-to-person interactions. In January 2020, Lai et al. estimated that the mean basic reproduction number (*R*_0_) of the virus was between 2 and 3.5 at that time in China; these reproduction numbers represent low and high levels of contagiousness, respectively. Velavan and Meyer (2020) reported that the average incubation time of the virus is ~ 6 days, and that it has three principal symptoms: fever, a dry cough, and shortness of breath. Guarner (2020) highlighted the role of SARS-CoV-2 in the pneumonia outbreak. Several vaccines have since been developed for SARS-CoV-2. These are based on the inactivated virus, RNA, DNA, nonreplicating and replicating viral vectors, and protein subunits (Dong et al. 2020). The most promising of the 180 vaccines that are at various stages of development are NVX-CoV2373 from Novavax, ChAdOx1 nCoV-19 from Oxford/AstraZeneca, and BNT162b1 and BNT162b2 from Pfizer/BioNTech (Krammer 2020). In addition, several drugs and therapeutic treatments have been used with varying success to alleviate the pneumonia caused by the novel coronavirus, including remdesivir (in the USA; Zhu et al. 2020), Arbidol (in Russia; Deng et al. 2020), favipavir (in Japan; Shiraki and Daikoku 2020), hydroxychloroquine (Gautret et al. 2020), and convalescent plasma donated by recovered patients (Shen et al. 2020; Zhang et al. 2020; Liu et al. 2020a). The fatality rates reported by different studies range from 2 to 5% (Jung et al. 2020; Li et al. 2020a; Schröder 2020).

The primary action taken by most governments around the world to contain the virus has been to lock down cities and other localities in which SARS-CoV-2 infections have been recorded. Furthermore, real-time monitoring and identification of infected people have been implemented by public institutions and health authorities to track the spread of the virus. This highlights the notion that the learning process early during an unknown virus outbreak is a crucial influence on the effectiveness of decisions made to tackle the outbreak (Lai et al. 2020; Naddeo 2020).

WHO has suggested that personal protective equipment (PPE) such as a long-sleeved gown, gloves, boots, a mask, and goggles or a face shield should be used for individual protection against SARS-CoV-2. Furthermore, WHO identified the use of a bleach solution (2–10% sodium hypochlorite) as an actionable method of disinfecting urban areas potentially contaminated with SARS-CoV-2.

The various transmission pathways of the novel coronavirus are currently being identified. This review summarizes current knowledge on SARS-CoV-2 transmission pathways and highlights the need to pay more attention to currently underestimated factors, such as indoor spread and outdoor risk sources. It is hoped that the points raised in this review will help governmental institutions and other cognizant authorities in their decision-making during the management of viruses outbreak.

## Knowledge of actual dispersion and transmission pathways

WHO has reported that this respiratory disease can be transmitted through respiratory droplets (particle diameter > 5–10 μm) and droplet nuclei (particle diameter < 5 μm) (WHO 2020). Droplet nuclei can remain suspended in the air and travel for long distances depending on the turbulence and flow of the air (WHO 2014). In general, airborne particles of size < 5 μm may remain suspended for hours depending on the air flow rate, turbulence, temperature, and humidity (Seto 2015). As reported by Li et al. (2020b) and Leung et al. (2020), an aerosol with particles of size > 10—20 μm can travel through the air for a short time and for a distance of < 1 m. Zhao et al. (2019) highlighted that aerosol size, air velocity, temperature, humidity, and flow rate are the main variables that significantly influence the dispersion of airborne pathogens.

Coronaviruses with sizes ranging from 0.05 to 0.20 μm are single-stranded RNA viruses with crown-like spike proteins on their surfaces (Wang et al. 2020a, b). These viruses can be aerosolized in droplet nuclei 5–10 μm in size (Leung et al. 2020). They are also non-lipid-membrane viruses, meaning that they are highly stable at high relative humidity (70–90%) (Wu et al. 2020a, b). Temperature can affect the states of viral proteins (including enzymes) and the virus genome. As reported by Pastorino et al. (2020) and Rabenau et al. (2005), temperatures above 60 °C decrease coronavirus infectivity in vitro, and maintaining this temperature for more than 30 min generally leads to SARS-CoV-2 inactivation. The surrounding layer of organic material acts as a moderate shield, protecting the virus from other environmental factors and hence increasing its infectivity for a long period. However, the temperature and humidity of its environment affect the transmissibility of the virus. Wang et al. observed that while high temperature and high humidity can both reduce the effective reproductive number (*R* value) associated with the spread of the virus, the *R* value cannot be decreased to less than unity (*R* < 1)—in which case the epidemic would die out gradually—by applying these measures (high temperature and high humidity) alone (Wang et al. 2020a, b).

**Fig. 1:**
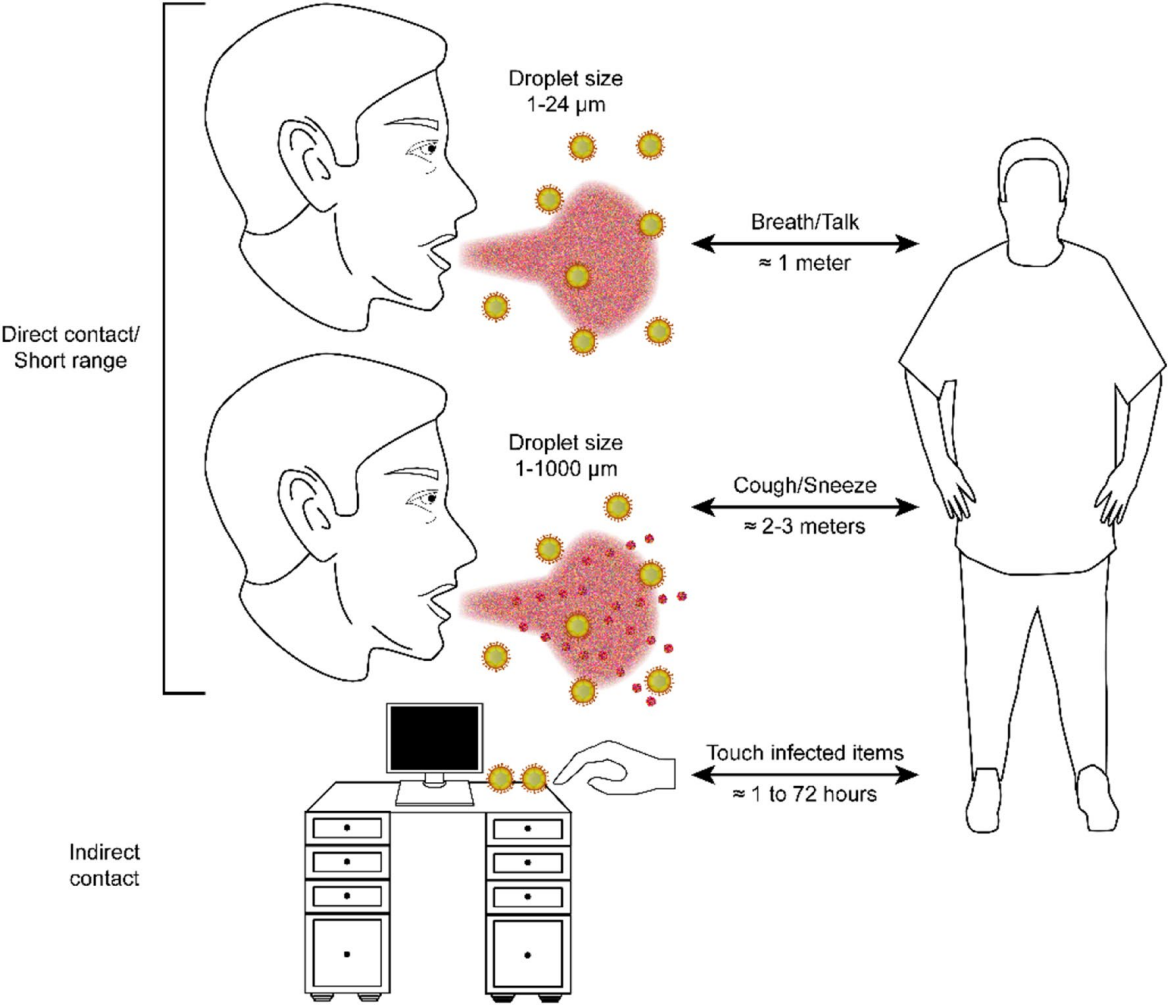
Dispersion and transmission pathways for SARS-CoV-2

Infected patients generate infectious droplets of varying sizes through breathing, coughing, or sneezing (WHO 2020). They also produce fecal matter that is rich in SARS-CoV-2 viruses (Wu et al. 2020a, b). As reported by the ISS (Istituto Superiore della Sanità, Italy), up to 200 droplets with diameters ranging from 1 to 24 μm can be emitted during conversations and breathing; these droplets spread over a distance of ~ 1 m (Bonadonna et al. 2020). This result was determined experimentally by a standardized method that involves measuring the droplets emitted by a person as they count the numbers from 1 to 100 out loud (Duguid 1946). Coughing and sneezing can generate up to 350,000 droplets with diameters ranging from 1 to 1000 μm that spread over a distance of ~ 2–3 m. Figure 1 summarizes these observations and presents other confirmed dispersion and transmission pathways of SARS-CoV-2 that are associated with direct and/or indirect contact.

Many studies have demonstrated that the spread and transmissibility of SARS-CoV-2 are much greater in indoor environments, especially in hospitals, laboratories, and schools, due to the presence of or proximity to viral sources as well as the increased possibility of direct contact with infected people or items (Huang et al. 2020). It was initially believed that coronaviruses cannot survive outdoors until Van Doremalen et al. (2020) found viable viral particles 3 h after they had been aerosolized. Van Doremalen et al. tested the viability of SARS-CoV-2 on different solid surfaces; their results showed that the virus was more stable on plastic and stainless steel than on copper and cardboard. Specifically, viable viruses were detected after 72, 24, and 4 h on plastic and steel, cardboard, and copper surfaces, respectively. Experimental tests were carried out at room temperature (22 °C) and a relative humidity of ~ 40%.

## Underestimated indoor spread and boost factors

Indoor environments in universities, schools, prisons, health care facilities, assisted living organizations, and daycare centers are areas where the spread and transmissibility of the novel coronavirus can be really high due to the large number of individuals present, the frequency of close interactions, and the likelihood of touching potentially contaminated items (Dietz et al. 2020). People spend more than 90% of their time in closed environments, so the possibility of being infected in them is potentially high.

Underestimated virus spread and boost factors in indoor environments are often associated with ventilation/filtration air systems. La Rosa et al. (2013) reported that it was possible for the virus to spread between passengers seated > 10 m apart in an airplane.

Park et al. (2020) identified a cluster of COVID-19 cases in a call center located in Seoul (South Korea), where 94 (43.5%) of the workers tested positive for SARS-CoV-2. Virus transmission between these workers likely occurred through direct and indirect contact with infected objects and persons and via short- and long-distance dispersion boosted by the ventilation system.

Lu et al. (2020) examined the transmission of the novel coronavirus in a restaurant without natural air circulation, where the clients were seated > 1 m apart. This study found that the main reason for the transmission of the virus in the artificial air flow was the air conditioner, which enabled the infectious droplets to travel for distances of > 1 m. This was confirmed by the observation that the people seated in the direction of the air stream were infected.

Table 1 summarizes the findings of a number of recent case studies in which heating, ventilation, and air conditioning (HVAC) systems were found to have significant potential to spread SARS-CoV-2 and boost its transmission.

**Table 1:** Case studies of SARS-CoV-2 infections that were facilitated by HVAC systems

Indoor environment	Description	Reference(s)
Restaurant (Guangzhou, China)	Infected clients were in the direction of the air flow from the air conditioner in a space without windows	Lu et al. (2020), Li et al. (2020b)
Hospital No. 1 (Wuhan, China)	SARS-CoV-2 RNA was detected in the air and on various items	Guo et al. (2020)
Hospital No. 2 (Wuhan, China)	35.7% of air outlets tested positive for SARS-CoV-2 RNA	Liu et al. (2020b)
Call center (South Korea)	46% of workers who shared the same office floor tested positive	Park et al. (2020)

**Fig. 2:**
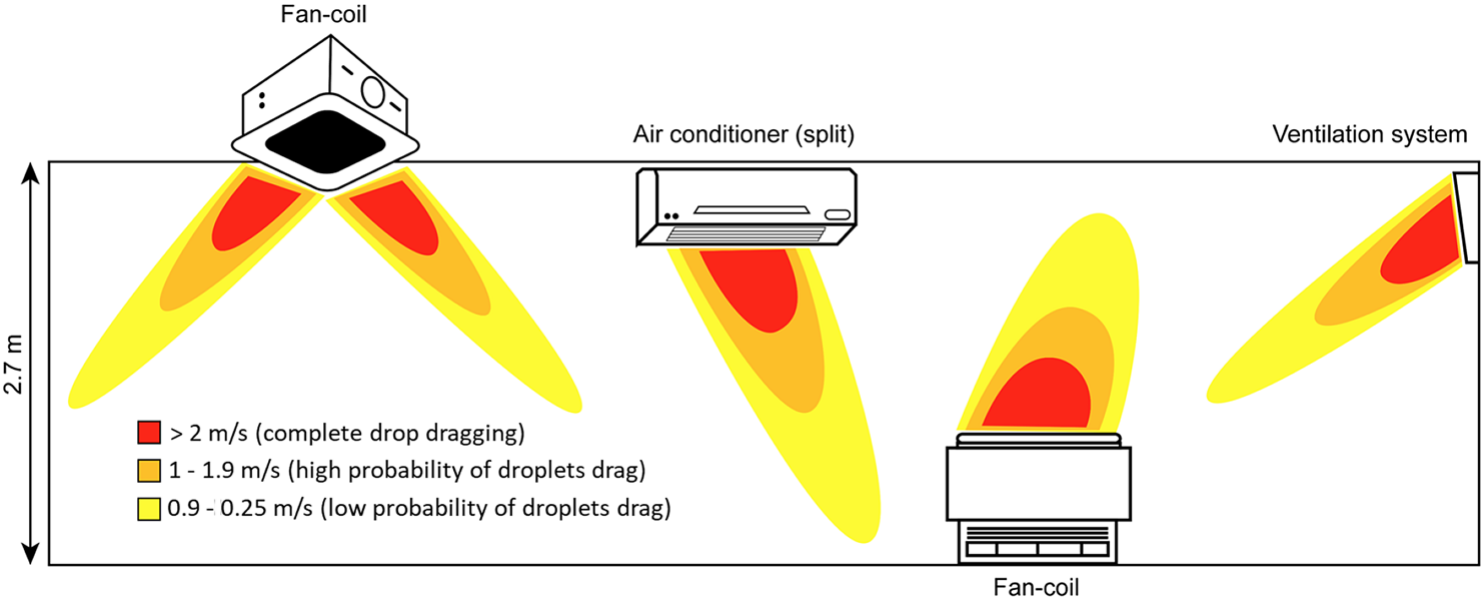
Droplet dragging by a HVAC system Adapted from Bonadonna et al. (2020)

As reported by Bonadonna et al. (2020), depending on the type of HVAC system considered, the distance and the velocity of the spread can lead to three different zones with different probabilities of droplet dragging (Fig. 2). Velocities above 2 m/s will drag droplets, whilst there is a high probability that droplets will be dragged when the velocity is between 1 and 1.9 m/s. There is a low probability that droplets will be dragged when the velocity is between 0.9 and 0.25 m/s.

To reduce the risk of infection in indoor environments, any HVAC system without an efficient air filtration apparatus should be upgraded. Efficient management (e.g periodically cleaning and sanitation) of the ventilation/filtration system should be done regularly to stop pathogens from persisting on the surface of the filter. Increasing natural ventilation, not lingering directly under the air flow, and minimizing spaces that are shared by people could help to minimize the spread of the virus in indoor environments.

## Underestimated outdoor risk sources

The long-distance transmission of viruses in outdoor environments has been confirmed by several studies (Andersen et al. 2009; Zhao et al. 2019). Van Doremalen et al. (2020) found that SARS-CoV-2 can be transmitted via aerosols, as the virus can remain viable and infectious for hours in an aerosol, and for days on surfaces. Andersen et al. (2009) reported that smoke and dust can contribute to the dispersion of viruses over long distances. Setti et al. (2020) found a high correlation (*R*^2^ = 0.98) between the number of new coronavirus infections in the north of Italy and the concentration of particulate matter ≤ 10 µm in diameter (PM_10_) in the air. This relationship would seem to indicate that fine dust particles can act as a carrier for the SARS-Cov-2 virus, boosting its spread and viability. This finding, if confirmed, implies that it is important to consider the involvement of novel outdoor environmental factors in SARS-CoV-2 dissemination, and thus to adopt adequate control actions.

Outdoor environments may lead to a higher risk of infection than indoor environments due to a lack of knowledge regarding the importance of outdoor environments as potential pathways for SARS-CoV-2 infection, the presence of large numbers of people in such environments, and the high likelihood of contact with contaminated elements or items. It is therefore essential to identify any risk factors in outdoor environments in order to prevent the spread of infection. Among these risk factors, wastewater may have an underestimated high potential for SARS-CoV-2 transmission (Naddeo and Liu 2020). Korzeniewska (2011) highlighted how wastewater can be a potential hotspot for the spread of viruses, in particular enteric viruses, because wastewater can provide an excellent environment for the growth of microorganisms. Gundy et al. (2009) proved that a coronavirus can survive in wastewater for periods ranging from a few hours to 2–3 days, depending on the temperature and the chemical composition of the wastewater. Many viruses have been shown to be present in wastewater (Corpuz et al. 2020), including an adenovirus (Kuo et al. 2010), a norovirus (Grøndahl-Rosado et al. 2014), and hepatitis E virus (La Rosa et al. 2010), which can become aerosolized due to their small sizes (< 1 µm). However, the viability of SARS-CoV-2 in wastewater has not been proven, so further studies are needed to investigate its fate in wastewater in detail. Masclaux et al. (2014) found up to 2 × 10^6^ viral particles/m^3^ in air samples in areas surrounding the treatment units in wastewater treatment plants (WWTPs). Wu et al. (2020a, b) found SARS-CoV-2 viruses in fecal samples, while Casanova et al. (2009) demonstrated that fecally contaminated liquid droplets are a potential vehicle for the spread of this virus. A number of studies have documented the presence of novel coronavirus RNA in wastewater (Ahmed et al. 2020; Alpaslan Kocamemi et al. 2020; Nemudryi et al. 2020; Randazzo et al. 2020; Rosa et al. 2020; Wu et al. 2020a, b; Wurtzer et al. 2020) (Table 2). A computational model developed by Hart and Halden (2020) estimated that the abundance of SARS-COV-2 in wastewater at 20 °C decreased by > 99% over the course of 2–3 days. Chin et al. (2020) demonstrated that SARS-CoV-2 can remain active and detectable in viral transport medium for 14 and 2 days at 22 and 37 °C, respectively. Conversely, Patel et al. (2020) reported that SARS-CoV-2 remains active for 25 days in wastewater at 5 °C. Finally, Bivins et al. (2020) determined that SARS-CoV-2 persisted in wastewater at room temperature for approximately 6 days.

**Table 2:** Studies that have detected the presence of SARS-CoV-2 in wastewater

Locale	Positive rate	Maximum concentration (RNA copies/L)	Reference
Queensland, Australia	20% (2/9)	1 × 10^2^	Ahmed et al. (2020)
Paris, France	100% (23/23)	3 × 10^6^	Wurtzer et al. (2020)
Milan, Italy	50% (6/12)	–	Rosa et al. (2020)
Monza, Italy	75% (3/4)	–	Rimoldi et al. (2020)
Murcia Region, Spain	11% (2/18)	1 × 10^5^	Randazzo et al. (2020)
Istanbul, Turkey	100% (7/7)	2.8 × 10^5^	Alpaslan Kocamemi et al. (2020)
Montana, USA	100% (7/7)	3 × 10^4^	Nemudryi et al. (2020)
Massachusetts, USA	71% (10/14)	2 × 10^5^	Wu et al. (2020a, b)

Hospital fecal discharges represent a very important potential source of SARS-Cov-2 transmission, due to the presence of a large number of infected patients. Where sewers are uncovered and open, they could be a major source of SARS-CoV-2 transmission, primarily due to the formation of contaminated aerosolized particles. Specific actions such as covering the sewers and using appropriate disinfection treatments and aerosolized air control systems can be highly effective and should therefore be urgently adopted, especially for these “open” sources, in order to limit or completely suppress their ability to infect and mass contaminate. In this context, it is notable that WWTPs treat their effluents using physical/chemical and/or biological processes (Muñoz et al. 2015; Senatore et al. 2020) but they do not inactivate or disinfect aerosolized virus particles. One possible strategy to minimize the risk posed by aerosolized particles is to implement an air treatment unit (e.g., an ozone generator or UV lamps) that effectively inactivates airborne viruses, including the novel coronavirus. SARS-CoV-2 is vulnerable to solar radiation and is effectively inactivated by UV radiation (Nicastro et al. 2020; Ratnesar-Shumate et al. 2020; Sagripanti and Lytle 2020). UV-B (*λ* = 280–315 nm) and UV-A (*λ* = 315–400 nm) radiation are able to penetrate through the Earth’s atmosphere to some extent, and could be an important factor in the fight against the SARS-CoV-2 virus in geographical areas where solar radiation is abundant, such as in the Mediterranean region (Sagripanti and Lytle 2020).

## Future perspectives and challenges

HVAC systems, aerosolized particles from wastewater, and particulate matter (PM) have been identified as underestimated infection pathways for the SARS-CoV-2 virus. These pathways can boost and spread COVID-19 transmission in indoor and outdoor environments, so they require careful consideration when attempting to limit the spread of the virus. Specific actions must be implemented by regulatory bodies to account for these risk factors, as they cannot be completely eliminated by social distancing alone. Further research into indoor and outdoor air quality—specifically, into measurement and monitoring strategies for controlling various pathogens—must be conducted to prevent and counter SARS-CoV-2 transmission and other potential biological risks. For the same reason, pathways that allow the virus to spread via solid waste and wastewater sector operations need to be carefully investigated. Infectious PPE waste management is another serious challenge to any governmental and/or other public health agency, given the large volumes of PPE waste that are expected to result from the COVID pandemic. The challenge is therefore to find alternative solutions for managing and disposing of used PPE.

## Abbreviations

COVID-19: Coronavirus Disease 2019
HVAC: Heating, ventilation, and air conditioning
ISS: Istituto Superiore della Sanità
MERS: Middle East Respiratory Syndrome
PPE: Personal protective equipment
SARS: Severe Acute Respiratory Syndrome
SARS-CoV-2: Severe Acute Respiratory Syndrome Coronavirus 2
WHO: World Health Organization
WWTP: Wastewater treatment plant
PM: Particulate matter
